# How are work engagement and workplace psychological safety related to sleep complaints in Japanese elementary school teachers? A nationwide cross‐sectional study

**DOI:** 10.1002/pcn5.70198

**Published:** 2025-09-03

**Authors:** Masateru Matsushita, Schuhei Yamamura

**Affiliations:** ^1^ Department of Psychology, Faculty of Psychology Konan Women's University Kobe Japan; ^2^ Department of Psychiatry Kinki Central Hospital of the Mutual Aid Association of Public School Teachers Itami Japan

**Keywords:** elementary school teachers, long working hours, sleep complaints, work engagement, workplace psychological safety

## Abstract

**Aim:**

Teachers experience a heavy workload and mental stress, which contributes to significant sleep problems. This study investigated the relationships between sleep complaints (sleep induction, awakenings during the night, poor overall quality of sleep, and sleepiness during the day), work engagement, and workplace psychological safety in public elementary school teachers in Japan.

**Methods:**

A cross‐sectional online survey was conducted among 96,421 elementary school teachers in Japan. Participants reported on their sleep complaints, work engagement, workplace psychological safety, and demographic and work‐related characteristics (age, sex, and years of experience, working hours per day). Multivariate logistic regression analyses were conducted to examine the relationships between work engagement, workplace psychological safety, and sleep complaints.

**Results:**

The study confirmed the high prevalence of sleep complaints among Japanese elementary school teachers. Both work engagement and workplace psychological safety were negatively associated with sleep complaints. Specifically, teachers working more than 13 h a day were significantly more likely to report complaints related to sleep induction and awakenings during the night. After adjusting for the confounding effects of working hours, the associations between work engagement, workplace psychological safety, and sleep complaints remained statistically significant.

**Conclusion:**

The findings suggest that addressing teachers' sleep complaints requires not only alleviating workload but also fostering a supportive organizational environment that promotes work engagement and workplace psychological safety. These insights can inform the development of policies and support measures to improve teachers' working conditions and overall well‐being.

## INTRODUCTION

Sleep plays a critical role in maintaining and promoting physical health, emotional stability, and cognitive performance.^1^, ^2^, ^3^ Adequate and restorative sleep contributes to individuals' quality of life and daytime functioning. Sleep complaints, including sleep induction, awakenings during the night, and excessive daytime sleepiness resulting from insufficient sleep duration, are often observed in the working population. These issues are associated with adverse mental health outcomes and diminished work productivity.^4^, ^5^, ^6^

In Japan, elementary school teachers perform various duties, including teaching, classroom management, parent communication, and school administrative tasks.^7^ Teaching is a helping profession, entailing significant emotional labor for educators.^8^ Furthermore, teachers usually work long hours.^9^, ^10^ Owing to a demanding work environment, teachers may experience both qualitative and quantitative sleep problems. Indeed, domestic studies report that approximately 40% of teachers complain of some form of insomnia symptoms, and long working hours and commuting times are reported as contributing factors.^11^ Despite the potential impact of teachers' sleep problems on not only their health but also the quality of education, epidemiological studies on sleep among teachers in Japan remain scarce. Further accumulation of relevant knowledge, including the investigation of related factors, is required.

Factors influencing sleep include individual stress responses and personality traits, with increasing attention being accorded to the psychosocial work environment.^12^ Work engagement, particularly, is highlighted as a positive psychological factor in the workplace. Work engagement is defined as a positive, fulfilling, work‐related state of mind characterized by three dimensions: vigor, dedication, and absorption.^13^, ^14^, ^15^ Similarly, workplace psychological safety is another crucial positive workplace factor. Psychological safety refers to a shared belief within a team that one can express their opinions and emotions without fear of interpersonal risk.^16^, ^17^ Studies have shown that a high level of workplace psychological safety promotes the exchange of ideas and mutual support, contributing to stress reduction.^18^ Although previous research has extensively examined the impact of negative factors such as mental stress and workload on sleep,^19^, ^20^ the investigation into the association between positive workplace factors and sleep remains scarce.

Few studies have investigated the issues of working hours and sleep among elementary school teachers. Furthermore, research on the association between positive workplace factors such as work engagement and workplace psychological safety and sleep, particularly among public school teachers, is limited. Given that prolonged working hours are closely related to shorter sleep duration, it is necessary to control for their effect when examining the association between work engagement, workplace psychological safety, and sleep in this population.

This study aims to (1) clarify the actual status of sleep complaints among public elementary school teachers throughout Japan and (2) examine the association between work engagement, workplace psychological safety, and sleep complaints.

## METHODS

### Participants and procedure

The subjects of this study included 101,339 elementary school teachers across Japan who had participated in a stress check program conducted by the Public School Mutual Aid Association at the request of municipal education boards nationwide. Data were collected online between June and December 2024. After excluding 4918 participants outside the age range of 21–61 years, 96,421 full‐time teachers remained for the final analysis. Only full‐time teachers were included in this study; those in administrative positions (e.g., principals, vice principals), part‐time or temporary teachers, school nurses, and nutrition teachers were excluded.

The survey collected information on age, sex, years of employment, job stress,^21^ type of school, the number of teaching staff at the school, job position and role, whether or not the participant has assignment as a homeroom teacher, average daily working hours in the past month, sleep complaints (sleep induction, awakenings during the night, overall quality of sleep, and sleepiness during the day), work engagement, workplace psychological safety, and stress factors.

To achieve the aim of this study, data on age, sex, the number of teaching staff at the participant's school, whether the participant had an assignment as a homeroom teacher, average daily working hours, sleep complaints, work engagement, and workplace psychological safety were utilized.

This study was conducted with the approval of the Kinki Central Hospital Research Ethics Committee (approval number: 500). The data were anonymized and provided in a de‐identified form, in accordance with the Declaration of Helsinki, and it was determined that written informed consent was not required.

### Measurements

#### Demographic characteristics

Participants were asked to provide responses to the following basic information: age (continuous variable), sex (men/women), number of teaching staff at the current school (1–24, 25–49, 50–99, and 100 or more), whether or not the participant was a homeroom teacher (yes/no), and average daily working hours (less than 8 h, 8 to less than 9 h, 9 to less than 10 h, 10 to less than 11 h, 11 to less than 12 h, 12 to less than 13 h, and 13 h or more). As only nine teachers worked at schools with 100 or more staff, this group was merged with the 50–99 staff group for the purpose of analysis.

#### Sleep complaints

Sleep complaints were assessed by asking participants about “sleep induction,” “awakenings during the night,” “overall quality of sleep,” and “sleepiness during the day,” with responses recorded on a 4‐point Likert scale. For multivariate logistic regression analysis, these responses were recategorized into a binary format: “problematic” (slightly problematic, markedly problematic, and extremely serious problem) scored as 1, and “no problem” (no problem or satisfactory) scored as 0.

#### Work engagement

Work engagement was assessed using two items: “At work, I feel like I am bursting with energy” [vigor] and “I am proud of the work that I do” [dedication]. Responses were rated on a 4‐point Likert scale: “Agree” (4 points), “Somewhat agree” (3 points), “Somewhat disagree” (2 points), and “Disagree” (1 point). The scores for the two items were summed and divided by 2 to calculate the average score. Higher scores indicate better work engagement. This simplified evaluation using two items has been reported to provide an equivalent assessment to the 9‐item version of the Utrecht Work Engagement Scale (UWES‐9).^22^

#### Workplace psychological safety

Workplace psychological safety was assessed using a single item: “In our workplace, we understand and acknowledge each other.” Responses were rated on a 4‐point Likert scale (4 = agree, 1 = disagree). Higher scores are interpreted as indicating a respectful and supportive workplace environment.

### Statistical analysis

For between‐group comparisons of continuous variables, a *t*‐test was conducted, and Cohen's *d* was calculated as the effect size. The criteria for effect size were as follows: *d* = 0.2 as small, *d* = 0.5 as medium, and *d* = 0.8 as large. For between‐group comparisons of categorical variables, a *χ*² test was conducted, and the *φ* coefficient was calculated as the effect size. The criteria for *φ* were defined as follows: *φ* = 0.1 as small, *φ* = 0.3 as medium, and *φ* = 0.5 as large. To assess relationships, Spearman's rank correlation coefficient (Spearman's *ρ*) was calculated.

To examine the relationships between each sleep complaint (sleep induction, awakenings during the night, overall quality of sleep, and sleepiness during the day), work engagement, and workplace psychological safety, multiple logistic regression analysis was performed. Age, sex, number of teaching staff at the school, whether the participant was a homeroom teacher, and average daily working hours were entered as covariates, and work engagement and workplace psychological safety were entered as independent variables to predict each sleep complaint. The forced entry method was employed for variable inclusion.

IBM SPSS Statistics for Windows, Version 23.0 (IBM Corp., Armonk, NY) was utilized for the analysis. In all statistical tests, the significance level was set at *p* < 0.05 for two‐tailed tests.

## RESULTS

The characteristics of the survey participants are presented in Table 1. A statistically significant difference was identified in age, with an effect size of 0.25, which is considered small; this difference indicated that women were slightly older than men. Significant differences were also observed between men and women in other variables; however, the effect sizes for these differences were all less than 0.1, indicating negligible effects. Regarding sleep complaints, 66.0% of the participants reported “no problem” with sleep induction, 65.1% reported “no problem” with awakenings during the night, 32.0% reported “no problem” with overall quality of sleep, and 23.8% reported “no problem” with sleepiness during the day.

**Table 1:** Comparisons of characteristics of participants between men and women.

	Total		Men		Women		*t*/*χ* ^2^	*p*	Effect size
Age, mean (SD)	40.2	(11.46)	38.4	(10.74)	41.2	(11.74)	−38.8	<0.001***	0.25
Number of faculty members, *n* (%)									
1–24	31,685	(32.9%)	12,298	(33.7%)	19,387	(32.4%)	26.7	<0.001***	0.02
25–49	55,302	(57.4%)	20,813	(57.0%)	34,489	(57.6%)			
50 or more	9434	(9.8%)	3400	(9.3%)	6034	(10.1%)			
Homeroom teacher status, *n* (%)									
Without	15,114	(15.7%)	6022	(16.5%)	9092	(15.2%)	29.8	<0.001***	0.02
With	81,307	(84.3%)	30,489	(83.5%)	50,818	(84.8%)			
Working hours/day, *n* (%)									
Less than 8 h	4102	(4.3%)	1220	(3.3%)	2882	(4.8%)	372.3	<0.001***	0.06
8–9 h	11,487	(11.9%)	4917	(13.5%)	6570	(11.0%)			
9 to less than 10 h	24,687	(25.6%)	9603	(26.3%)	15,084	(25.2%)			
10 to less than 11 h	22,296	(23.1%)	8070	(22.1%)	14,226	(23.7%)			
11 to less than 12 h	20,425	(21.2%)	7283	(19.9%)	13,142	(21.9%)			
12 to less than 13 h	9338	(9.7%)	3646	(10.0%)	5692	(9.5%)			
13 h and more	4086	(4.2%)	1772	(4.9%)	2314	(3.9%)			
Work engagement, mean (SD)	2.9	(0.67)	2.9	(0.71)	2.9	(0.64)	11.1	<0.001***	0.08
Workplace psychological safety, mean (SD)	3.1	(0.70)	3.1	(0.72)	3.1	(0.68)	5.0	<0.001***	0.03
Sleep induction, *n* (%)									
No problem	63,665	(66.0%)	24,386	(66.8%)	39,279	(65.6%)	25.3	<0.001***	0.02
Slightly delayed	26,232	(27.2%)	9665	(26.5%)	16,567	(27.7%)			
Markedly delayed	4663	(4.8%)	1707	(4.7%)	2956	(4.9%)			
Very delayed or did not sleep at all	1861	(1.9%)	753	(2.1%)	1108	(1.8%)			
Awakenings during the night, *n* (%)									
No problem	62,727	(65.1%)	23,721	(65.0%)	39,006	(65.1%)	108.9	<0.001***	0.03
Minor problem	26,980	(28.0%)	9978	(27.3%)	17,002	(28.4%)			
Considerable problem	5611	(5.8%)	2242	(6.1%)	3369	(5.6%)			
Serious problem or did not sleep at all	1103	(1.1%)	570	(1.6%)	533	(0.9%)			
Overall quality of sleep, *n* (%)									
Satisfactory	30,883	(32.0%)	12,014	(32.9%)	18,869	(31.5%)	49.0	<0.001***	0.02
Slightly unsatisfactory	50,522	(52.4%)	18,670	(51.1%)	31,852	(53.2%)			
Markedly unsatisfactory	11,246	(11.7%)	4277	(11.7%)	6969	(11.6%)			
Very unsatisfactory or did not sleep at all	3770	(3.9%)	1550	(4.2%)	2220	(3.7%)			
Sleepiness during the day									
None	22,977	(23.8%)	7817	(21.4%)	15,160	(25.3%)	694.6	<0.001***	0.08
Mild	60,734	(63.0%)	22,649	(62.0%)	38,085	(63.6%)			
Considerable	11,242	(11.7%)	5245	(14.4%)	5997	(10.0%)			
Intense	1468	(1.5%)	800	(2.2%)	668	(1.1%)			

*** *p* < 0.001.

Table 2 presents the results of the correlation analysis between each sleep complaint and work engagement as well as workplace psychological safety. Statistically significant negative correlations were observed between work engagement and all sleep complaints: sleep induction (*ρ* = −0.231, *p* < 0.001), awakenings during the night (*ρ* = −0.227, *p* < 0.001), overall quality of sleep (*ρ* = −0.318, *p* < 0.001), and sleepiness during the day (*ρ* = −0.264, *p* < 0.001). Similarly, workplace psychological safety was negatively correlated with all sleep complaints: sleep induction (*ρ* = −0.192, *p* < 0.001), awakenings during the night (*ρ* = −0.189, *p* < 0.001), overall quality of sleep (*ρ* = −0.249, *p* < 0.001), and sleepiness during the day (*ρ* = −0.169, *p* < 0.001). All these correlations were statistically significant, with the strength of the association ranging from small to moderate. These findings indicate that higher levels of work engagement and workplace psychological safety were associated with fewer sleep complaints.

**Table 2:** Associations between work engagement, workplace psychological safety, and sleep complaints.

	1. WE		2. WPS		3. SI		4. ADN		5. OQS		6. SDD	
	*ρ*	*p*	*ρ*	*p*	*ρ*	*p*	*ρ*	*p*	*ρ*	*p*	*ρ*	*p*
1. Work engagement		‐	0.424	<0.001***	−0.231	<0.001***	−0.227	<0.001***	−0.318	<0.001***	−0.264	<0.001***
2. Workplace psychological safety				‐	−0.192	<0.001***	−0.189	<0.001***	−0.249	<0.001***	−0.169	<0.001***
3. Sleep induction						‐	0.427	<0.001***	0.421	<0.001***	0.178	<0.001***
4. Awakenings during the night								‐	0.486	<0.001***	0.184	<0.001***
5. Overall quality of sleep										‐	0.334	<0.001***
6. Sleepiness during the day												‐

Abbreviations: ADN, awakenings during the night; OQS, overall quality of sleep; SI, sleep induction; SDD, sleepiness during the day; WE, work engagement; WPS, workplace psychological safety.

*** *p* < 0.001.

Table 3 presents the results of a multiple logistic regression analysis, with each sleep complaint as the dependent variable, work engagement and workplace psychological safety as the independent variables, and age, sex, number of teaching staff at the current school, homeroom teacher status, and working hours as covariates.

**Table 3:** Relationships among sleep complaints, work engagement, and psychological safety in the workplace among elementary school teachers.

	Sleep induction_(with/without)_				Awakenings during the night_(with/without)_				Overall quality of sleep_(with/without)_				Sleepiness during the day_(with/without)_			
	Wald	*p*	OR	95% CI	Wald	*p*	OR	95% CI	Wald	*p*	OR	95% CI	Wald	*p*	OR	95% CI
Age	109.92	<0.001***	1.01	1.01–1.01	1546.56	<0.001***	1.03	1.02–1.03	933.99	<0.001***	1.02	1.02–1.02	110.75	<0.001***	0.99	0.99–0.99
Sex																
Male	*Reference*															
Female	0.54	0.463	1.01	0.98–1.04	56.00	<0.001***	0.90	0.87–0.92	17.14	<0.001***	0.94	0.91–0.97	265.62	<0.001***	0.76	0.74–0.79
Number of faculty members																
1–24	*Reference*															
25–49	5.12	0.024*	1.04	1.00–1.07	14.62	<0.001***	1.06	1.03–1.09	10.28	0.001 **	1.05	1.02–1.09	2.58	0.108	1.03	0.99–1.06
50 or more	3.41	0.065	1.05	1.00–1.10	2.18	0.139	1.04	0.99–1.09	3.69	0.055	1.05	1.00–1.11	0.00	0.988	1.00	0.95–1.06
Homeroom teacher status																
Without	*Reference*															
With	2.75	0.097	0.97	0.93 −1.01	0.23	0.630	1.01	0.97–1.05	0.24	0.624	0.99	0.95–1.03	105.98	<0.001***	0.79	0.75–0.83
Working hours/day																
Less than 8 h	*Reference*															
8 to less than 9 h	0.00	0.973	1.00	0.93–1.08	2.78	0.096	0.94	0.86–1.01	0.06	0.800	0.99	0.92–1.07	0.00	0.952	1.00	0.92–1.08
9 to less than 10 h	0.96	0.328	1.04	0.96–1.12	1.08	0.298	0.96	0.89–1.04	20.43	<0.001***	1.18	1.10–1.27	9.44	0.002**	1.13	1.05–1.22
10 to less than 11 h	0.01	0.911	1.00	0.93–1.08	3.71	0.054	0.93	0.86–1.00	78.80	<0.001***	1.40	1.30–1.51	25.43	<0.001***	1.22	1.13–1.32
11 to less than 12 h	0.75	0.388	1.03	0.96–1.11	0.62	0.430	0.97	0.90–1.05	154.38	<0.001***	1.61	1.50–1.74	51.26	<0.001***	1.34	1.24–1.45
12 to less than 13 h	1.24	0.265	1.05	0.97–1.14	0.01	0.933	1.00	0.92–1.08	264.40	<0.001***	2.01	1.85–2.19	90.99	<0.001***	1.55	1.42–1.69
13 h and more	6.21	0.013*	1.13	1.03–1.24	6.20	0.013*	1.13	1.03–1.24	231.31	<0.001***	2.23	2.01–2.47	80.06	<0.001***	1.65	1.48–1.84
Work engagement	2490.73	<0.001***	0.56	0.55–0.57	2521.47	<0.001***	0.56	0.54–0.57	4216.01	<0.001***	0.42	0.41–0.43	2978.20	<0.001***	0.46	0.44–0.47
Workplace psychological safety	942.09	<0.001***	0.71	0.70–0.73	858.11	<0.001***	0.72	0.71–0.74	1026.57	<0.001***	0.67	0.66–0.69	270.79	<0.001***	0.81	0.78–0.83

*Note*: Responses on the sleep complaints were dichotomized as “with” (slightly problematic, markedly problematic, and extremely serious problem) and “without” (no problem at all).

Abbreviations: 95% CI, 95% confidence interval; OR, odds ratio.

*** *p* < 0.001

** *p* < 0.01

* *p* < 0.05.

Sleep induction was significantly associated with age (Wald = 109.92, *p* < 0.001, odds ratio [OR] = 1.01, 95% confidence interval [CI] = 1.01–1.01), the number of teaching staff at the current school (25–49: Wald = 5.12, *p* = 0.024, OR = 1.04, 95% CI = 1.00–1.07), average daily working hours (13 h or more: Wald = 6.21, *p* = 0.013, OR = 1.13, 95% CI = 1.03–1.24), work engagement (Wald = 2490.73, *p* < 0.001, OR = 0.56, 95% CI = 0.55–0.57), and workplace psychological safety (Wald = 942.09, *p* < 0.001, OR = 0.71, 95% CI = 0.70–0.73). Consistent with the correlation analysis, higher work engagement and workplace psychological safety were associated with lower odds of sleep induction (OR < 1).

Awakenings during the night were associated with age (Wald = 1546.56, *p* < 0.001, OR = 1.03, 95% CI = 1.02–1.03), sex (women: Wald = 56.00, *p* < 0.001, OR = 0.90, 95% CI = 0.87–0.92), the number of teaching staff at the current school (25–49: Wald = 14.62, *p* < 0.001, OR = 1.06, 95% CI = 1.03–1.09), average daily working hours (13 h or more: Wald = 6.20, *p* = 0.013, OR = 1.13, 95% CI = 1.03–1.24), work engagement (Wald = 2521.47, *p* < 0.001, OR = 0.56, 95% CI = 0.54–0.57), and workplace psychological safety (Wald = 858.11, *p* < 0.001, OR = 0.72, 95% CI = 0.71–0.74). Consistent with the correlation analysis, higher work engagement and workplace psychological safety were associated with lower odds of awakenings during the night (OR < 1).

Overall quality of sleep was associated with age (Wald = 933.99, *p* < 0.001, OR = 1.02, 95% CI = 1.02–1.02), sex (women: Wald = 17.14, *p* < 0.001, OR = 0.94, 95% CI = 0.91–0.97), the number of teaching staff at the current school (25–49: Wald = 10.28, *p* = 0.001, OR = 1.05, 95% CI = 1.02–1.09), average daily working hours (9 to less than 10 h: Wald = 20.43, *p* < 0.001, OR = 1.18, 95% CI = 1.10–1.27; 10 to less than 11 h: Wald = 78.80, *p* < 0.001, OR = 1.40, 95% CI = 1.30–1.51; 11 to less than 12 h: Wald = 154.38, *p* < 0.001, OR = 1.61, 95% CI = 1.50–1.74; 12 to less than 13 h: Wald = 264.40, *p* < 0.001, OR = 2.01, 95% CI = 1.85–2.19; 13 h or more: Wald = 231.31, *p* < 0.001, OR = 2.23, 95% CI = 2.01–2.47), work engagement (Wald = 4216.01, *p* < 0.001, OR = 0.42, 95% CI = 0.41–0.43), and workplace psychological safety (Wald = 1026.57, *p* < 0.001, OR = 0.67, 95% CI = 0.66–0.69). Consistent with the correlation analysis, higher work engagement and workplace psychological safety were associated with lower odds of poor quality of sleep (OR < 1).

Sleepiness during the day was associated with age (Wald = 110.75, *p* < 0.001, OR = 0.99, 95% CI = 0.99–0.99), sex (women: Wald = 265.62, *p* < 0.001, OR = 0.76, 95% CI = 0.74–0.79), whether the participant was a homeroom teacher (Wald = 105.98, *p* < 0.001, OR = 0.79, 95% CI = 0.75–0.83), average daily working hours (9 to less than 10 h: Wald = 9.44, *p* = 0.002, OR = 1.13, 95% CI = 1.05–1.22; 10 to less than 11 h: Wald = 25.43, *p* < 0.001, OR = 1.22, 95% CI = 1.13–1.32; 11 to less than 12 h: Wald = 51.26, *p* < 0.001, OR = 1.34, 95% CI = 1.24–1.45; 12 to less than 13 h: Wald = 90.99, *p* < 0.001, OR = 1.55, 95% CI = 1.42–1.84; 13 h or more: Wald = 80.06, *p* < 0.001, OR = 1.65, 95% CI = 1.48–1.84), work engagement (Wald = 2978.20, *p* < 0.001, OR = 0.46, 95% CI = 0.44–0.47), and workplace psychological safety (Wald = 270.79, *p* < 0.001, OR = 0.81, 95% CI = 0.78–0.83). Consistent with the correlation analysis, higher work engagement and workplace psychological safety were associated with lower odds of sleepiness during the day (OR < 1).

## DISCUSSION

This study aimed to clarify the relationships between sleep complaints (sleep induction, awakenings during the night, overall quality of sleep, and sleepiness during the day), work engagement, and workplace psychological safety among elementary school teachers in Japan. Data from a large‐scale cross‐sectional survey involving approximately 96,000 participants were analyzed using multiple logistic regression analysis. The results indicated that both work engagement and workplace psychological safety were negatively associated with various sleep complaints. In other words, the improvement of the workplace environment may contribute to enhancing sleep quality in teachers.

The findings of this study align with those of previous research on general workers,^23^, ^24^ supporting the notion that environments characterized by high work engagement and psychological safety may enhance the quality of sleep. While relevant to all helping professions, work engagement plays a significant role in reducing stress and fostering positive emotions, especially among teachers.^25^, ^26^ Moreover, since teachers must share student information and coordinate events with colleagues, workplace psychological safety plays a crucial role in reducing stress and fostering teamwork. Consequently, work engagement and psychological safety may be associated with improved sleep quality by mitigating stress.

When teachers have high work engagement, find their jobs fulfilling, feel motivated, and are fully immersed, they experience less mental stress. Generally, lower stress levels help the mind relax, leading to better sleep quality, including easier sleep onset. A psychologically safe workplace allows teachers to express their opinions freely and collaborate with their colleagues, creating a stable and supportive work environment. Such an environment alleviates anxiety and tension, ultimately promoting more restorative sleep. Consequently, this can lead to a more positive attitude towards work and enhanced mental stability, potentially resulting in improved sleep, including easier sleep induction and fewer awakenings during the night. Future research could yield significant insights by exploring how to boost work engagement and enhance workplace psychological safety in the workplace.

Conversely, negative workplace factors, especially prolonged working hours, also had a significant impact on teachers' sleep. Regarding the relationship between working hours and sleep, working more than 13 h per day was significantly associated with sleep induction and awakenings during the night. Both difficulty falling asleep and frequent awakenings are symptoms of insomnia. A workday of 13 h or more corresponds to more than 100 h of overtime per month. Previous studies involving teachers have reported that working more than 13 h per day is associated with physical and mental stress responses,^27^ suggesting that excessive workloads may be linked to insomnia symptoms. Chronic insomnia symptoms increase the risk of depression.^5^ Therefore, addressing overwork among teachers is crucial for mental health prevention.

Working more than nine hours a day was closely linked to poorer sleep quality and increased daytime sleepiness. Moreover, the longer the work hours, the more pronounced the daytime drowsiness became. These findings suggest that both poor quality of sleep and sleepiness during the day are more likely to reflect sleep deprivation rather than insomnia. This implies that extended working hours may curtail total sleep time, resulting in poorer subjective quality of sleep and diminished daytime alertness. Sleep deprivation increases the risks of hypertension or diabetes.^2^ Furthermore, as noted by Stenson et al., chronic sleep deprivation not only impairs memory and learning abilities but also disrupts emotional regulation.^28^ Therefore, the potential impact of prolonged working hours on the quality of education should not be underestimated.

In summary, the burden of insomnia or sleep deprivation associated with teachers working extended hours is not merely a personal health issue; it can also impair teacher performance, potentially compromising the overall standard of education at the school.

The novelty of this study lies in its focus on a specific population—elementary school teachers across Japan—and in the concurrent examination of both work engagement and psychological safety in relation to multiple aspects of sleep complaints. Use of relatively simple measurements about sleep complaints facilitated a nationally representative survey, which enhanced the generalizability of the findings. The results have important implications for teacher health support policies, suggesting that improving teachers' sleep issues may require not only a reduction in workload but also a systemic workplace improvement initiative aimed at enhancing work engagement and workplace psychological safety. The initiatives of individual teachers are insufficient to mitigate negative workplace factors such as prolonged working hours and excessive workload. Fundamental improvements require considerable time and organizational support. However, positive workplace factors, namely, work engagement and workplace psychological safety, can be enhanced through the efforts of teachers and school administrators. As improving workplace psychological safety is a key managerial responsibility, it can yield relatively immediate outcomes.

There are several limitations to this study. First, as this is a cross‐sectional study, causal relationships cannot be inferred. Second, this study did not investigate personal life stressors such as parenting or caregiving responsibilities. To enhance teacher well‐being, it is crucial to examine the impact of various stress factors on sleep. Third, for the evaluation of sleep complaints, a brief measure was employed. Future use of validated scales such as the Athens Insomnia Scale could potentially provide results with higher reliability and validity. Fourth, the measurement of working hours relied on self‐reports, which should also be considered as a potential limitation. Future research should include objective sleep indicators, such as actigraphy, and longitudinal data to explore the temporal changes in workplace factors and sleep problems.

In conclusion, this study clarified that work engagement and workplace psychological safety are related to sleep complaints among Japanese elementary school teachers. The findings suggest that improving organizational environments could be an effective measure to address sleep problems in educational settings. Future efforts to develop teacher support strategies should examine the importance of enhancing workplace environments.

## AUTHOR CONTRIBUTIONS

Masateru Matsushita and Schuhei Yamamura conceptualized this study. Masateru Matsushita analyzed the data and wrote the manuscript. Schuhei Yamamura supervised the study. Both authors contributed to manuscript revision and approved the submitted version.

## CONFLICT OF INTEREST STATEMENT

The authors declare no conflicts of interest.

## ETHICS APPROVAL STATEMENT

The studies involving human participants were reviewed and approved by the Ethics Committee of the Kinki Central Hospital of the Mutual Aid Association of Public School Teachers (Approval No. 500).

## PATIENT CONSENT STATEMENT

Written informed consent for participation was not required for this study in accordance with the National Legislation and the Institutional Requirements.

## CLINICAL TRIAL REGISTRATION

N/A.

## Data Availability

The data that support the findings of this study are available on request from the corresponding author. The data are not publicly available due to privacy or ethical restrictions.
